# Anesthetic Approach in Ambulatory Vitrectomy: Peribulbar Block vs. Balanced General Anesthesia

**DOI:** 10.1155/2022/3838222

**Published:** 2022-03-28

**Authors:** Bárbara Gouveia, Leonardo Ferreira, Paula Maia

**Affiliations:** Department of Anesthesiology, Centro Hospitalar Universitário de São João, Porto, Portugal

## Abstract

**Background:**

Vitrectomy is one of the most common outpatient ophthalmic surgeries. The anesthetic technique used in outpatient surgery should contribute to a faster functional recovery, better pain control, and fewer complications. The aim of this study was to compare peribulbar block and balanced general anesthesia, in patients undergoing outpatient vitrectomy.

**Methods:**

A prospective cohort study was carried out, including adult patients undergoing ambulatory vitrectomy, between January and February 2018. Peribulbar block or balanced general anesthesia was the independent variable analyzed. Clinical and perioperative variables were evaluated, namely, postoperative pain, nausea, and vomiting in the postoperative period, intraoperative hypotension, patient satisfaction with the anesthetic technique, time to oral diet introduction and to hospital discharge, operating room occupancy time, and pharmacological costs. SPSS^®^ 27 was used for statistical analyses.

**Results:**

Twenty-one patients were evaluated, 11 of whom underwent peribulbar block and 10 underwent balanced general anesthesia. Patients undergoing peribulbar block did not experience postoperative pain when compared to patients undergoing balanced general anesthesia (*p*=0.001). Intraoperative hypotension occurred in 18.2% of patients undergoing peribulbar block and in 70% of those undergoing balanced general anesthesia (*p*=0.03). Time to oral diet introduction (<1 hour vs. > 2 hours; *p* < 0.05), operating room occupancy time (70 vs. 90 minutes; *p*=0.027), time to hospital discharge (17 vs. 22.5 hours; *p*=0.004), and pharmacological costs (4.65 vs. 12.09 euros; *p* < 0.05) were lower in patients undergoing peribulbar block versus balanced general.

**Conclusions:**

Peribulbar block seems to meet the criteria of an ideal anesthetic technique in outpatient vitrectomy surgery.

## 1. Introduction

Outpatient surgery is expanding over the last decade due to inclusion of surgical procedures increasingly complex [1, 2].

Vitrectomy is one of the most common outpatient ophthalmic surgeries [3]. There is no evidence of the best type of anesthesia for this ambulatory surgery, but usually, it is performed under general or regional anesthesia [3]. The anesthetic technique used in outpatient surgery should contribute to a faster functional recovery, better pain control, lesser use of opioids, fewer complications, and lower economic costs.

The aim of this study was to compare peribulbar block and balanced general anesthesia, in patients undergoing outpatient vitrectomy.

## 2. Materials and Methods

**Figure 1:**
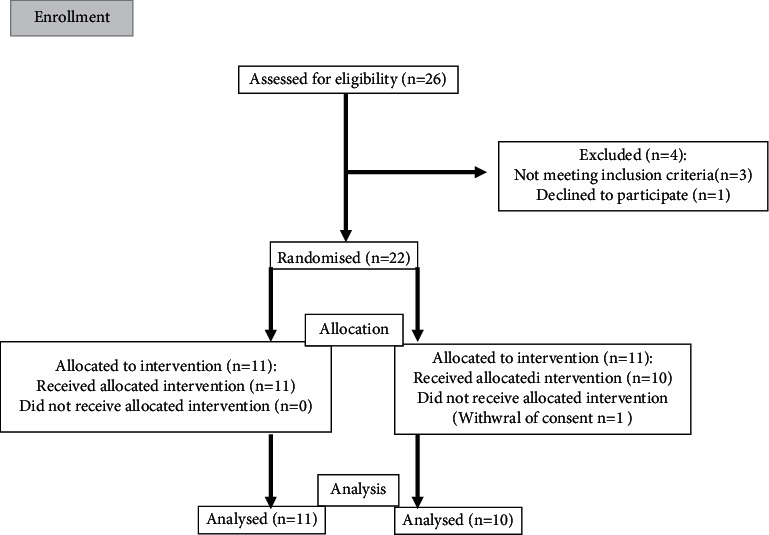
Flow diagram of the study participants.

After approval by the ethics committee (protocol number 150–2018), a prospective cohort study was carried out, including adult patients undergoing outpatient vitrectomy, between January and February 2018. Patients were randomized to receive balanced general anesthesia or peribulbar block. Those under 18 years or with any contraindication to peribulbar block were excluded (Figure 1).

The following variables were analyzed: gender, age, American Society of Anesthesiologists physical status, postoperative pain, nausea, and vomiting in the postoperative period. Intraoperative hypotension, patient satisfaction with the anesthetic technique, time to oral diet introduction, operating room occupancy time, time to hospital discharge, and pharmacological costs were analyzed. The type of anesthesia, peribulbar block, or balanced general anesthesia was the independent variable analyzed.

Twenty-one patients were evaluated, and data were subjected to statistical treatment, Mann–Whitney, chi-square, and Fisher's exact tests, and *p* values < 0.05 were considered statistically significant. SPSS^®^ 27 was used for statistical analyses.

All patients were monitored with ASA standard monitoring.

In the peribulbar block group, 4% oxybuprocaine eye drops were placed, and 5 minutes after, an inferotemporal percutaneous prick with G25 1 inch needle was performed. After aspiration, 3–5 ml of the anesthetic solution (4 ml ropivacaine 0.75% and 2 ml lidocaine 2%) was injected. Ocular compression balloon (Honan balloon) was placed, after the block, with 30–35 mmHg pressure. Ten minutes later, the balloon was removed, and ocular akinesia was evaluated. In patients who did not obtain ocular akinesia, a second prick was made in the superonasal or caruncular region with a G25 8 inch needle, and 2-3 ml of the anesthetic solution was injected.

Patients submitted to general anesthesia were induced with fentanyl 2 mcg/kg and propofol 2 mg/kg, and a supraglottic device was inserted. Maintenance of anesthesia was achieved with a mixture of oxygen, air, and sevoflurane, adjusted to maintain anesthetic depth, based on bispectral index values between 40 and 60.

For postoperative analgesia, 1 g of paracetamol and 30 mg of ketorolac were administered 30 minutes before the end of surgery.

## 3. Results

Twenty-one patients were evaluated, 11 of whom underwent peribulbar block and 10 underwent balanced general anesthesia.

The main results are given in Table 1. Patients undergoing peribulbar block did not experience postoperative pain when compared to patients undergoing balanced general anesthesia (*p*=0.001).

Intraoperative hypotension occurred in 18.2% of patients undergoing peribulbar block and in 70% of those undergoing balanced general anesthesia (*p*=0.03). Time to oral diet introduction was less than 1 hour in those under peribulbar block versus more than 2 hours in patients under balanced general anesthesia (*p* < 0.05). Operating room occupancy time (average 70 vs. 90 minutes; *p*=0.027), time to hospital discharge (17 vs. 22.5 hours; *p*=0.004), and pharmacological costs (4.65 vs. 12.09 euros; *p* < 0.05) were lower in patients undergoing peribulbar block versus balanced general anesthesia.

Of the 11 patients submitted to peribulbar block, 4 had chemosis, which spontaneously reversed, without other associated complications.

There were no statistically significant differences in the variables ASA physical status, nausea, and vomiting in the postoperative period and patient satisfaction in relation to the anesthetic technique, regarding the type of anesthesia.

## 4. Discussion

General anesthesia and peribulbar, retrobulbar, and subtenon blocks are the anesthetic techniques most consistently described for vitreoretinal surgery [3, 4].

Although general anesthesia is the most anesthetic technique historically used for this procedure, Licina et al. argue that local anesthesia, in the form of topical or injectable application of a local anesthetic, has gained prominence in recent years as an anesthetic technique for surgery involving the vitreous and retina [3].

However, the literature remains controversial as to the ideal anesthetic technique for performing this intervention [3]. Furthermore, despite knowing that vitrectomy is one of the most common outpatient ophthalmic surgeries, there is little literature regarding which anesthetic technique may be more suitable for this surgery regime [3].

In our study, we found that none of the patients anesthetized with peribulbar block reported pain in the postoperative period. However, of the patients undergoing general anesthesia, 7 reported pain, requiring recourse to opioids. This fact seems to be due to the prevention of central hyperexcitability by the noxious stimulus, which happens due to the decrease in the afferent stimulus of muscle traction, obtained through the peribulbar block [5, 6].

Other studies have shown that patients submitted to vitreo and retinal surgery with peribulbar block and general anesthesia experience less postoperative pain compared to patients undergoing only general anesthesia [5, 6]. This study adds that peribulbar block as a single anesthetic technique is effective in controlling postoperative pain in the first 6 hours, without opioids.

Decreased use of opioids and consequent reduced time to hospital discharge are some of the benefits described in the literature of peripheral nerve blocks in ambulatory surgery [7]. In our work, we also showed that in outpatient vitrectomy, the choice of peribulbar block as an anesthetic technique allows the patient to have an earlier discharge when compared to general anesthesia (17 vs. 22 hours; *p*=0.004), which can be explained by the good pain control with better postoperative recovery.

The costs related to peripheral nerve blocks are mainly attributed to the blocking catheters and infusion pumps/syringes used when performing a continuous nerve block [7]. In this case, we are facing a single shot blockade, so the costs are only related to drugs, syringes, needles, and other necessary materials. In addition, we showed that drug costs associated with peribulbar block were significantly lower (median 4.65 vs. 12.09 euros; *p* < 0.05) than drugs used in general anesthesia. This fact can be explained by the higher cost of intravenous and inhaled anesthetics, as well as the need for additional analgesic medication in the group undergoing general anesthesia.

In our study, there were no statistically significant differences in the prevalence of nausea and vomiting in patients undergoing each of the anesthetic techniques. This result contradicts previous studies, which argue that peribulbar block is associated with a decrease in postoperative nausea and vomiting, as it blocks the oculoemetic reflex [5, 8]. However, Moret et al. found that in patients undergoing retinal surgery, there were no differences in the incidence of nausea and vomiting within 24 hours postoperatively in patients undergoing general anesthesia and peribulbar block [9]. Our results can be explained by the fact that patients undergoing general anesthesia received propofol as an inducing agent, ondansetron for nausea and vomiting prophylaxis, and also because other emetic agents, such as nitrous oxide, were not administered.

Most patients presented for ophthalmic surgery are elderly patients, with associated comorbidities and whose anesthetic technique should maintain hemodynamic homeostasis [10]. In our study, we demonstrated that peribulbar block caused less hemodynamic changes, and only 18.2% had intraoperative hypotension with the need of vasopressors when compared to 70% in the group undergoing general anesthesia. As in the literature, more than 50% of patients were over 65 years of age, which is the most vulnerable group, in which hemodynamic instability may cause more deleterious consequences [10].

Beside chemosis that spontaneously reversed, we did not find any other type of complications associated with peribulbar block, such as optic nerve damage, hemorrhage, or eye perforation [11].

No difference was found between general and regional anesthesia with regard to surgical time, according to a meta-analysis by Liu et al. [12]. In our study, we demonstrated that operating room occupancy time was significantly shorter in patients undergoing peribulbar block compared to the group undergoing general anesthesia. Although initially peribulbar block may be technically more difficult and require more time, this time is recovered at the end of surgery, once there is no time needed to wake up the patient.

Ghali and El Btarny report that patient dissatisfaction, as well as discomfort associated with the surgical time and the need to remain immobile, are the main factors that limit the isolated use of regional anesthesia as an anesthetic technique for vitreous and retinal surgery [5]. Our study combats this idea, as patients anesthetized with peribulbar block were not less satisfied with the anesthetic technique than patients undergoing general anesthesia, making this criterion not valid to contraindicate a peribulbar block alone.

The introduction of oral feeding after general anesthesia requires the patient to be fully awake, with preserved airway reflexes, in order to reduce the risk of pulmonary aspiration [13]. This may be the justification for the fact that, in our study, patients undergoing peribulbar block were able to feed earlier postoperatively than patients undergoing general anesthesia.

Our study has a small sample, and so, despite our results showing advantages for regional anesthesia, it is important to perform more studies to verify and corroborate our results and conclusions.

Peribulbar block was associated with fewer intraoperative complications, shorter operating room occupancy, hospital stay, and oral diet introduction, with lower pharmacological costs and more effective pain control, without the need of opioids, when compared with general anesthesia, and for that, we concluded that peribulbar block seems to meet the criteria of an ideal anesthetic technique in outpatient vitrectomy surgery.

## Figures and Tables

**Table 1:** Results according to the type of anesthesia.

		*N* Peribulbar block	*N* Balanced general anesthesia	%	*P*
Postoperative pain at 1 h, 3 h, and 6 h	Yes	0	7	33.3	0.001
	No	11	3	66.7	
Intraoperative hypotension	Yes	9	3	57.1	0.03
	No	2	7	42.9	
Oral diet introduction time	<1 hour	11	0	52.4	0.000
	1-2 hours	0	0	0	
	2-3 hours	0	6	28.6	
	3-4 hours	0	4	19	
Operating room occupancy time	Mean	70.91 min	104.50 min		0.027
	Median	70 min	90 min		
	Minimum	45 min	60 min		
	Maximum	90 min	180 min		
Time to hospital discharge	Mean	15.18 h	21.8 h		0.004
	Median	17 h	22.5 h		
	Minimum	11 h	17 h		
	Maximum	24 h	24 h		
Pharmacological costs	Mean	5.45€	33.5€		0.004
	Median	4.65€	12.09€		
	Minimum	4.65€	5.4€		
	Maximum	9.06€	90.92€		

## Data Availability

The data used to support the findings of this study are included within the article.
